# Detection and Quantification of Precious Elements in Astrophyllite Mineral by Optical Spectroscopy

**DOI:** 10.3390/ma14216277

**Published:** 2021-10-21

**Authors:** Altaf Ahmad, Shahab Ahmed Abbasi, Muhammad Hafeez, Taj Muhammad Khan, Muhammad Rafique, Nasar Ahmed, Pervaiz Ahmad, Mohammad Rashed Iqbal Faruque, Mayeen Uddin Khandaker, Muhammad Javed

**Affiliations:** 1 Department of Chemistry, University of Azad Jammu and Kashmir, Muzaffarbad 13100, Pakistan; altafahmadmir@gmail.com (A.A.); muhammad.hafeez@ajku.edu.pk (M.H.); 2 Department of Physics, University of Azad Jammu and Kashmir, Muzaffarbad 13100, Pakistan; mrafique@ajku.edu.pk (M.R.); nasar.ahmed@ajku.edu.pk (N.A.); pervaiz.ahmad@ajku.edu.pk (P.A.); javedbinyousuf@gmail.com (M.J.); 4 School of Physics and CRANN, Trinity College Dublin, University of Dublin, Dublin 2, Ireland; 3 National Institute of Lasers and Optronics College, Pakistan Institute of Engineering and Applied Sciences, Nilore, Islamabad 45650, Pakistan; khanta@tcd.ie; 5 Space Science Centre, Universiti Kebangsaan Malaysia, UKM, Bangi 43600, Malaysia; 6 Center for Applied Physics and Radiation Technologies, School of Engineering and Technology, Sunway University, Bandar Sunway 47500, Malaysia; mayeenk@sunway.edu.my; 7 Department of Physics, University of Kotli, Kotli 11100, Pakistan

**Keywords:** CF-LIBS, astrophyllite, minerals, titanium, quantitative analysis

## Abstract

With many advantages over well-established methods, laser induced breakdown spectroscopy (LIBS) has emerged as a useful analytical technique for the compositional analysis of multi-elemental geological materials. In this study, LIBS was employed for qualitative and quantitative analysis of a rare mineral, astrophyllite, bearing precious elements of industrial and technological interest. The experiment was carried out using second harmonic generation of Nd:YAG laser of pulse width 5 ns and repetition rate of 10 Hz. Microplasma was produced by focusing laser beam on an astrophyllite target, and optical emissions from the generated plasma were recorded in the spectral range of 200–720 nm with the help of a LIBS2000+ detection system. On analyzing the optical spectra, existence of 15 elements in astrophyllite target were revealed. These elements include: Ti, W, Ag, Al, Ba, Ca, Cr, Cu, Fe, Li, Mg, Na, Ni, Si and H. For quantification, calibration-free method was used. Only ten elements, namely Ti, W, Fe, Cr, Cu, Ca, Mg, Ni, Si and Al, were quantified with relative weight concentrations of 55.39%, 18.79%, 18.30%, 4.05%, 2.66, 0.43%, 0.18%, 0.12%, 0.06% and 0.02%, respectively. To benchmark these results, XRF analysis was performed, which confirmed the presence of all the elements detected in the optical spectrum of the sample, except for Na, Li, and H. The concentrations of these ten elements as measured by XRF were in reasonable agreement, especially for the major elements. The presence of a significant amount of Ti and W in an astrophyllite sample, found in Pakistan, highlights the economic value of this mineral. This study may be of further interest in commissioning LIBS technology for exploration of minerals in the region.

## 1. Introduction

Elemental analysis of minerals containing precious elements has always been important from a commercial and technological point of view [1]. For the extraction of precious and semi-precious elements, reliable detection of these elements is of prime importance. Astrophyllite is rare and among one of the important Ti-bearing minerals [2,3]. The astrophyllite super group contains twelve different minerals and possesses topographical dependent compositional structure because of regional metamorphism [4,5,6]. Titanium (Ti) metal is ductile, malleable, half as dense as iron, twice as dense as Al, and has good luster, and low electrical and thermal conduction [7]. It possesses a hexagonal close-packed and body-centered crystal structure and has five naturally occurring isotopes, namely Ti-46, Ti-47, Ti-48, Ti-49 and Ti-50. By mixing with other metals such as aluminum, molybdenum and iron, alloys can be made with tensile strength greater than titanium itself. Formation of passive oxide on its surface makes it resistant in all types of environments [8]. It is as hard as steel but has much lower density as compared to steel. Due to low density and corrosion-resistant nature in extreme conditions, Ti-based alloys are the materials of choice for air- and spacecraft applications [9]. Alloys of Ti have also found applications in the biomedical field and are commonly used for hard tissue replacement, joint replacement and dental fillings, owing to its non-toxic nature [10,11]. Titanium possesses a higher refractive index and can absorb ultraviolet (UV) light; these properties make TiO_2_ a multi-function material. Nanomaterials of TiO_2_ have found interesting applications, such as photo catalytic degradation, electrochromic devices, photovoltaic cells, hydrogen storage, and sensing instruments [12].

For extraction of such an important metal such as Ti, reliable and efficient elemental detection in geological materials is highly desirable. Various analytical techniques commonly used for elemental quantification and qualitative analysis in minerals are energy dispersive X-ray spectroscopy (EDX), inductively coupled plasma mass spectrometry (ICP-MS), X-ray fluorescence (XRF), atomic absorption spectroscopy (AAS), proton induced X-ray emission (PIXE), electron probe micro-analyzer with wavelength dispersive spectrometers (EPMA-WDS), and Raman spectroscopy. Laser-induced breakdown spectroscopy (LIBS) has emerged as an alternative analytical technique, having many advantages over aforementioned techniques such as no specific sample preparation, simultaneous elemental identification, fast response, real-time and in situ analysis, detection of almost every element in the periodic table from light to heavy, analysis of different phases of material (liquid, solid and gas), ability of remote sensing, minimally destructive technique, contact free, and spatial- and depth-resolved analysis [13]. In LIBS analysis, the target is irradiated with a high-powered laser beam, resulting in melting and evaporation of a part of the irradiated material and converts it into a micro-plasma plume. Atoms and ions in the plasma plume form the excited states, and on cooling of the plasma, these atoms de-excited to ground states by emitting the characteristic spectral radiation. Each element has a unique spectral signature, and hence by proper identification of the emitted lines, the elements present in the target material can be accurately and precisely identified [14]. Due to the theoretical and technical advancements, LIBS technique is now an effective tool for the investigation of geological materials [15,16,17,18,19]. For the purpose of quantitative analysis of a multi-elemental sample, calibration and calibration-free laser-induced breakdown spectroscopy (CF-LIBS) methods are traditionally practiced. The calibration method requires calibration curves for all the compositional elements of a sample and comparison with the optical emission spectrum of the corresponding element [20,21]. The use of this method in quantitative analysis of multi-elemental geological samples is limited by the lengthy and tedious procedure and the error caused by the matrix effect [22,23], whereas the calibration-free method involves the measurement of elemental concentration of elements in a sample by theoretical calculations using values of the plasma parameters [24,25]. This methodology is preferred over the calibration method because it eliminates the matrix effect and requires no reference sample. CF-LIBS technique has been successfully employed for compositional analysis of various types of ores and minerals such as quartz, limestone and granite gneiss [26,27,28]. Concentration of Cu along with other elements has been successfully measured using CF-LIBS method in copper-rich minerals such as malachite and chalcopyrite [29,30]. For quantitative analysis, CF-LIBS technique has been implemented for the compositional analysis of meteorites and other extra-terrestrial objects [31,32].

In the current study, compositional analysis of Ti bearing astrophyllite minerals, of local origin, using LIBS, is presented. To the best of our knowledge, there is no report available online on the elemental analysis of an astrophyllite by LIBS. The primary objective of this study is to detect and quantify a precious and scientifically important element, Ti, in an astrophyllite mineral. This research work can be helpful in exploration of Ti as well as other elements from this region. Further, this study has potential to open new possibilities of using LIBS technology for the practical purpose of mining in the country.

## 2. Methodology

### 2.1. Study Area, Geology and Sampling

Astrophyllite mineral was collected from Leswa district Neelam of Azad Jammu and Kashmir, Leswa, Pakistan (geographical coordinates are 34°27′55″ N, 73°47′10″ E). This mineral is one of the accessory minerals of orthogenesis, and its formation is due to the regional metamorphism of granite. It is not a commonly found mineral, and its existence is restricted to only alkaline-type granites and is intruded into Salkhala formation. Orthogenesis lies in the lesser Himalayas, and its southern contact is with Salkhala formation, whereas northern contact is within Jura formation [33]. This region is mainly composed of chlorite schist, biotitic schist, garnet schist with the minor occurrence of graphitic schist along with marble [34].

Overall, ten pieces of astrophyllite, from different locations of the site containing the reserves, were collected. Quantitative analysis using CF-LIBS methodology involves careful theoretical calculations; hence, analysis of a large number of samples requires considerable time. Keeping this aspect in view, we ground all ten samples into powder form and took a small portion from all ten powdered samples and mixed it. Afterward, to obtain one representative sample, this mixture was pressed under 30 ton pressure for 20 min with a pellet presser, and in this way, a pellet of 20 mm diameter and 2 mm thickness was obtained. A part of the mixture was reserved for XRF analysis.

### 2.2. Experimental

The experimental arrangement for this study is also described elsewhere in detail [35,36,37]. In short, a plasma plume was generated on the sample surface by focusing a laser beam originating from a Q-switched Nd.YAG laser (Brilliant, Quintal, Les Ulis CEDEX, France). This laser has 5 ns plus duration and 10 Hz repetition rate with 450 mJ laser energy at 532 nm wavelength. For focusing of laser beam on astrophyllite target, a 20 cm quarts focusing lens was used. To prevent air breakdown, the lens to sample distance was kept shorter than the focal length of the lens. Plasma on the surface of the target was generated using a laser pulse having energy 120 mJ. Diameter of laser spot on the surface of target was 0.55 mm as measured with the help of an optical microscope. Calculated value of laser irradiance, using values of pulse energy and spot area for this experiment was 1.0 × 10^10^ W/cm^2^. An energy meter (Nova Quantel P/niz01507, France) was utilized to measure the laser pulse energy. The sample was placed on a rotatory stage to provide the fresh surface to every laser shot to minimize the effect of sample heterogeneity and avoid formation of deep craters on target surface. This rotation of target also helps in obtaining decent short-to-short signal reproducibility. Optical emission spectra were recorded using an optical fiber, connected with LIBS 2000+ system (Ocean Optics. Inc., Orlando, FL, USA), having a collimating lens with 0–45^o^ field of view and positioned orthogonally to the laser beam direction. The emission spectrum of each sample was generated at the optimized delay time (3 μs) and integration time (2.1 ms), because after 2 μs time delay, plasma is supposed to be sufficiently homogeneous. Optical signals from the plasma were recorded by LIBS2000+ detection system, comprising a set of five spectrometers.

**Figure 1:**
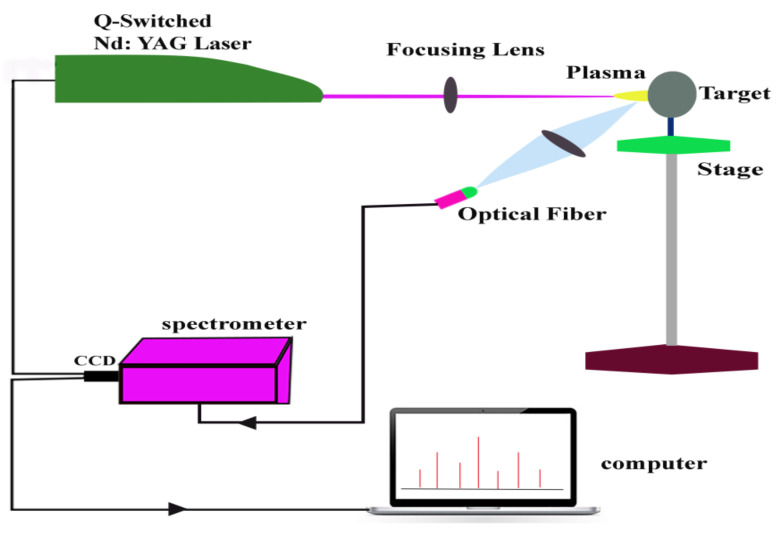
Schematic diagram of LIBS2000+ experimental setup.

This system was calibrated in efficiency using standard light source DH-2000-CAL (Ocean Optics, Inc. Orlando, FL, USA). Each spectrometer was outfitted with a 5 μm slit, 2048 element linear CCD array with a spectral resolution of ≈0.06 nm. The data presented in this paper were obtained by accumulating 10 laser shots for each averaged spectrum of the sample, which considerably increased the signal-to-noise ratio. The acquired data were stored on a computer by software for subsequent analysis. Schematic diagram of described experimental setup is presented in Figure 1.

## 3. Results and Discussion

The recorded optical emission spectrum of astrophyllite was subsequently analyzed qualitatively as well as quantitatively as described in the following sections.

### 3.1. Qualitative Analysis

**Figure 2:**
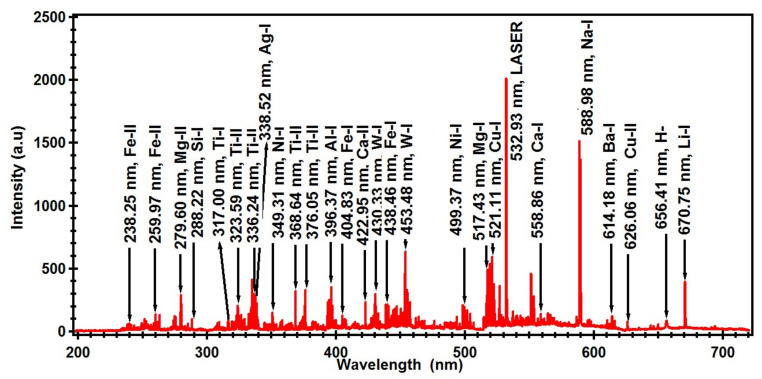
Obtained optical emission spectrum from laser-produced plasma of astrophyllite in the spectral range of 200–720 nm.

The LIBS spectrum of astrophyllite in the spectral range from 200 to 720 nm is shown in Figure 2.

**Figure 3:**
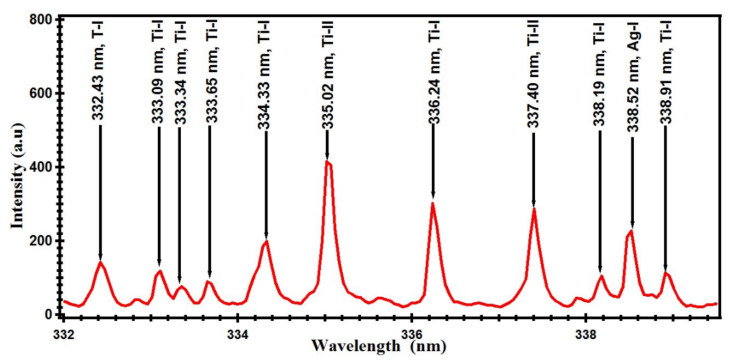
Optical emission spectrum of laser-produced astrophyllite plasma in the narrow spectral range of 330–340 nm.

Spectral lines of compositional elements of astrophyllite were identified by comparing wavelength values in the unit of nm from the spectrum with a spectroscopic database available online by the National Institute of Standard and Technology, Gaithersburg, MD, USA (NIST) [38]. Few of the prominent spectral lines of identified elements in the sample are labeled in the complete spectrum of astrophyllite (Figure 2). For the purpose of clarity for the readers, a part of the spectrum in the narrow h from 330 to 340 nm is presented in Figure 3. This region is dominated by the spectral Ti spectral lines emitted by neutral, Ti-I, and singly ionized, Ti-II atoms. Analysis has revealed that the astrophyllite spectrum contains emission lines of a variety of elements including Ti, W, Ag, Al, Ba, Ca, Cr, Cu, Fe, Li, Mg, Na, Ni, and Si. We detected 05 lines of silver (Ag), 04 lines of aluminum (Al), 08 lines of barium (Ba), 11 lines of calcium (Ca), 06 lines of copper (Cu), 05 lines of chromium (Cr), 50 lines of iron (Fe), 02 lines of lithium (Li), 08 lines of magnesium (Mg), 02 lines of sodium (Na), 13 lines of nickel (Ni), 05 lines of silicon (Si), 60 lines of titanium (Ti) and 22 lines of tungsten (W). All the identified lines of the above-mentioned elements are listed in Table 1.

### 3.2. Quantitative Analysis

After elemental identifications, for the compositional analysis, the CF-LIBS technique was utilized [24]. For this purpose, three basic prerequisite conditions are required to be fulfilled: (i) existence of local thermodynamic equilibrium (LTE); (ii) optically thin plasma; (iii) stoichiometric ablation [39,40].

#### 3.2.1. Electron Temperature

**Figure 4:**
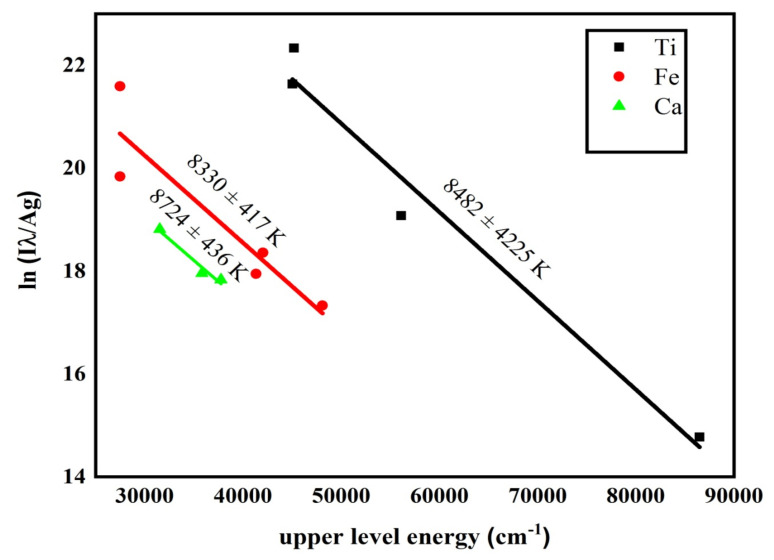
Boltzmann plot drawn for measurement of plasma temperature using spectral lines of Ti, Fe, and Ca.

The famous Boltzmann plot method was utilized to obtain the plasma temperature using the following expression [41].

(1) $$ln\left(\frac{I\lambda }{hcAg\left(k\right)}\right)=ln\left(\frac{N\left(t\right)}{U\left(t\right)}\right)-\frac{E_{k}}{KT_{e}}$$

where *I* is integrated line intensity of emission line, *λ* is the wavelength of line, *A* is transition probability, *g* is statistical weight of upper level (*k*), *N*(*t*) total number density, *U*(*t*) partition function, *E_k_* is upper-level energy, *K* is Boltzmann constant and *Te* is electron temperature. Plasma temperature was determined with ±5 percent error due to uncertainty in transition probability and measurement of the integrated intensity of selected lines. Boltzmann plot was drawn by using spectroscopic data of atomic emission lines of Ti-I at 323.59, 338.19, 363.74, 368.64, 400.14; Fe-I at 240.55, 373.86, 438.62, 441.77; and Ca-I at 445.33, 612.14, 646.33, which is shown in Figure 4. Spectroscopic data of used lines taken from the NIST database [34] are listed in Table 2. From the slope of the straight line, −1/*kT_e_*, obtained by plotting *ln* $\left(\frac{I\lambda }{hcAg}\right)$ against the upper-level energy *E_k_*, plasma temperature can be measured. Uncertainty of about ±5%, caused by uncertainty in the measurement of integrated line intensities and transition probabilities, arose in the measured value of temperature. Values of plasma temperature calculated from Boltzmann plot of neutral lines of Ti, Ca and Fe were 8482 ± 424, 8724 ± 436, and 8330 ± 417 K, respectively. The average temperature was 8512 ± 426 K, and this value was subsequently used for further calculations.

#### 3.2.2. Determination of Electron Number Density

For the estimation of electron number density (Ne) in the plasma, the stark broadened line profile technique was used. The number density depends upon the full width at half maximum (FWHM) of the stark broadened line as shown in the following expression [42,43].

(2) $$N_{e}=\frac{\Delta \lambda_{1/2}\times 10^{16}}{2\omega }$$

where $\Delta \lambda_{1/2}$ is FWHM and *ω* is a stark broadening parameter. The value of $\Delta \lambda_{1/2}$ was used after subtracting instrumental width, 0.06 nm, from the calculated value.

(3) $$\Delta \lambda_{1/2}=\Delta \lambda_{1/2}\left(Calculated\right)-\Delta \lambda_{1/2}\left(Instrumental\right)$$

Lorentzian fitting of selected lines provided the values of FWHM and stark broadening parameter, ω, was obtained from data available online [44]. Calcium atomic lines at 616.22 and 646.33 nm and Si-I lines at 251.65 and 288.18 nm were selected for the calculation of electron number density. Calculated values of electron number densities are given in Table 3, and the average value was taken as$\;4.04\times 10^{16}{\mathrm{cm}}^{-3}$.

#### 3.2.3. Validity of Local Thermodynamic Equilibrium

For the existence of local thermodynamic equilibrium (LTE), atomic states must be populated and depopulated dominantly by electronic collision rather than by radiation. Mc-Whirter criterion provides a minimum value of electron number density required to ensure a high collision rate. This threshold value of n_e_ can be obtained using the following expression [21,45].

(4) $$Ne\left(cm^{-3}\right)\geq 1.6\times 10^{12}{\left(T_{e}\left(k\right)\right)}^{\frac{1}{2}}\;{\left(\Delta E\left(ev\right)\right)}^{3}$$

where $T_{e}$ electron temperature is ΔE is the energy difference between the involved energy states expected to be in LTE, and Ne is electron number density, respectively. It was found that the value of experimentally calculated Ne is much higher than the threshold value, as calculated from Equation (4); this may confirm that plasma under investigation satisfies the existence of condition of LTE. Lines from other elements, in the astrophyllite spectrum, viz. Fe, Ti, Cu, Ca, and Si, have also been tested and verified for the same criteria.

#### 3.2.4. Optically Thin Plasma

To validate the condition of optically thin plasma, intensity ratio method was employed. In this method, ratio of intensities of selected lines were calculated using this expression [46]:

(5) $$\frac{I_{1}}{I_{2}}=\frac{\lambda_{2}A_{1}g_{1}}{\lambda_{1}A_{2}g_{2}}exp\left(-\frac{E_{1}-E_{2}}{K_{B}T}\right)$$

where $I_{1}\;\mathrm{and}\;I_{2}$ are the integrated intensities of selected lines as calculated from Lorentzian fitting, whereas $E_{1}\;\mathrm{and}\;E_{2}$ are the energies of the upper level of transitions involving these lines.

In this method, experimental and theoretical intensity ratio of those lines are compared which share the same upper level energy or their upper level energy values are close to each other. This selection criteria results in reducing the exponential factor on right hand side of Equation (5) to unity. A pair of lines of Ca-I at 616 and 646 nm was chosen carefully, and the experimental and theoretical intensity ratio of selected lines was found to be 0.82 and 0.73, respectively, which are in close agreement. This result verified that plasma was optically thin.

#### 3.2.5. Stoichiometric Ablation

Condition of stoichiometry ablation is that the value of laser irradiance at the target surface should be greater than$\;10^{9}\;Wcm^{-2}$ [47]. In this experiment, the calculated value of laser irradiance was 1.0 × 10^10^ $\mathrm{Wc}m^{-2}$, which shows that laser ablation was definitely stoichiometric.

#### 3.2.6. Calibration-Free Analysis

For quantification of astrophyllite sample, several emission lines of different elements identified in emission spectra were selected based on selection criteria laid down in reference [25,48,49]. Selected lines of various elements in the sample such as Cr, Cu, Fe, Ti, and W are listed in Table 4. Emission lines of Ag, Li, and Na could not pass the selection criteria, and hence these elements could not be quantified.

Atomic number density of neutral atoms from different elements in plasma were calculated using Equation (6).

(6) $$N_{x}^{i}=\frac{I\lambda U_{x}^{i}\left(T\right)}{A_{ki}g_{k}}\;\left(4\times 10^{14}\right)e^{\frac{E_{k}}{K_{B}T}}$$

where $E_{k}$ is the energy of the upper level and $U_{x}^{i}\left(T\right)$ is the partition function of the neutral atom, and other parameters are the same as mentioned previously.

The Saha equation was used for calculation of number density of singly ionized atoms [47,48]:

(7) Nxii=2 NXiNe−16.02×1021Te32UxiiTUxiTe^−xiKBT

where, $N_{x}^{i}$, $U_{x}^{ii}\left(T\right),\;and\;x_{i}$ are number density of neutral atoms, the partition function of singly ionized atoms at measured temperature $T_{e}$ and ionization potential of element respectively. By summing the number density of neutral and singly ionized atoms, as calculated from Equations (6) and (7), the total relative atomic number density of the element was obtained.

The relative weight of each element in the sample was calculated by multiplying the atomic weight of each element with its corresponding relative atomic number density ($WA_{x}\times N_{x}$). The elemental weight thus obtained was added together and then the corresponding sum$\;\sum_{x}^{\;}(WA_{x}\times N_{x})$, was used to determine the weight percentage of the compositional element by using the following expression [47,48,49]:

(8) $$W\%N_{x}=\frac{WA_{x}\times N_{x}}{\sum_{x}WA_{x}\times N_{x}}\times 100$$

**Figure 5:**
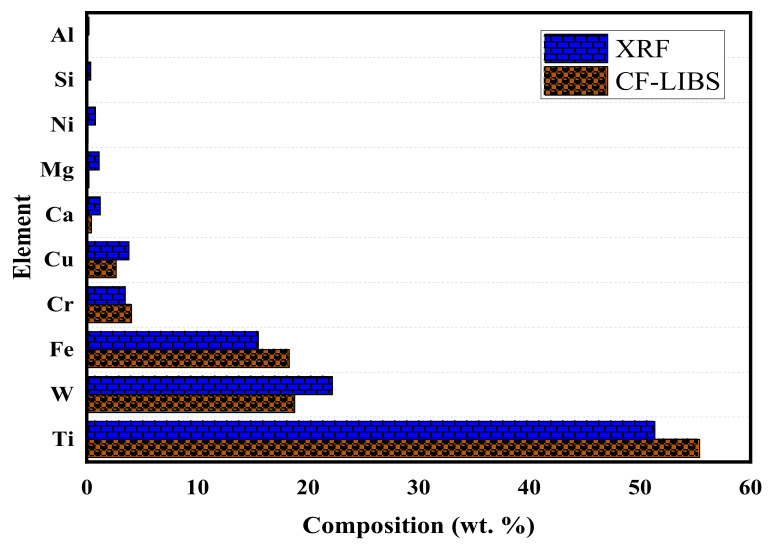
Elemental composition of astrophyllite as measured by CF-LIBS and XRF.

Calculated values of relative concentration of Ti, W, Fe, Cr, Cu, Ca, Mg, Ni, Si and Al in astrophyllite sample were 55.39%, 18.74%, 18.42%, 4.05%, 2.67%, 0.35%, 0.18, 0.12%, 0.06% and 0.02% by weight, respectively. To verify the quantitative results obtained from CF-LIBS analysis, X-ray fluorescence (XRF) technique was performed on the same sample. Before the analysis, energy calibration of XRF was carried out using JSX-3200 standard material. XRF analysis showed the concentration of all the elements quantified by CF-LIBS. The concentration of Ti, W, Fe, Cr, Cu, Ca, Mg, Ni, Si and Al as measured by XRF were 51.34%, 22.20%, 15.51%, 3.48%, 3.82%, 1.22%, 1.12%, 0.78%, 0.36% and 0.17% by weight, respectively. For the purpose of comparison, these quantitative results obtained by CF-LIBS and XRF are presented in Figure 5. The relative error in measurement of elemental concentration of the sample by both techniques is given in Table 5.

## 4. Discussion

The main structural unit of astrophyllite super group mineral is the H-O-H block and consists of three H–O–H sheets, where the T_4_O_12_ astrophyllite ribbons occur in the H sheets. In each structure, HOH blocks alternate with intermediate (I) blocks. The astrophyllite supergroup contains twelve subgroups of minerals with variable elemental compositions. To accommodate this compositional diversity, a generalized formula of these minerals in the form of A_2p_B_r_C_7_D_2_(T_4_O_12_)_2_ I$X_{D_{2}}^{O}X_{A_{4}}^{O}X{\;}_{D_{n}}^{P}W_{A2}$ was devised. In this general formula, C = Fe^2+^, Mn, Fe^3+^, Na, Mg or Zn; D = Ti, Nb, Zr, Fe^3+^; T = Si, Al; A_2p_B_r_I = is the composition of the I block, and subscript *p* = 1, 2; A = K, Cs, Li, Ba, H_2_O; B = Na, Ca, Ba, H_2_O; I denotes the composition of the central part of the I block, where *p* = 1, 2; r = 1, 2; $X_{D_{2}}^{O}X_{A_{4}}^{O}X{\;}_{D_{n}}^{P}$ = O, OH, F and H_2_O; n = 0, 1, 2; $W_{A2}$ = H_2_O. In order to describe atomic arrangements in the intermediate space between adjacent HOH blocks in the astrophyllite-group structures, an intermediate (I) block was introduced [2,3]. In LIBS analysis, we detected overall 15 elements, namely Ti, W, Ag, Al, Ba, Ca, Cr, Cu, Fe, Li, Mg, Na, Ni, Si, and H in the investigated sample. Except for W, Ag, Cr, Cu, and Ni, all other elements were present in the chemical formula of astrophyllite super group mineral. From detected compositional elements, we assume that the mineral under investigation resembled the subgroup, Bulgakite, more closely, with chemical formula Li_2_(Ca,Na)${\mathrm{Fe}}_{7}^{2+}$Ti_2_(Si_4_O_12_)_2_O_2_(OH)_4_(O,F)(H_2_O)_2_ [2].

Oxygen could not be detected in this experiment because its strong lines lie outside of the spectral range of our spectrometer. The low excitation efficiency of fluorine makes identification of fluorine with the LIBS hard, thus it was also not detected in the current study [36].

Astrophyllite is one of the rare minerals bearing precious and industrially and technologically important elements such as Ti, Fe, Cr, Cu, Ca, Mg, Ni, Si, Li, and Al. Using the CF-LIBS method, we quantified the elements detected in optical spectrum of astrophyllite as Ti (55.39%), W (18.74%), Fe (18.42%), Cr (4.05%), Cu (2.67%), Ca (0.35%), Mg (0.18), Ni (0.12%), Si (0.06%) and Al (0.02%), whereas H, Li, Na, Ba, and Ag could not be quantified by CF- LIBS methodology due to the unavailability of suitable lines fulfilling the required criteria. With more than 50% concentration of Ti, the investigated astrophyllite sample seemed to be a high-grade Ti-bearing mineral, which is a desirable property in the mineral industry for the extraction of Ti, which has found numerous scientific applications. Along with Ti, the astrophyllite sample also contained a significant amount of W, another scientifically important metal that enhances the economic value of this mineral further. In our study, measured concentration of Ti was more than 50%, which is higher than the concentration suggested by the chemical formula of this mineral. Similarly, the value of Si concentration was lower than expected values indicated by chemical formula. There can be many reasons for this anomaly, including: (i) regional metamorphism; (ii) heterogeneity of target sample; (iii) low amount of ablated mass (as optical spectrum represents the ablated material in the plasma plume); (iv) collected samples were only the small pieces of rock; thus it is possible that, due to heterogeneity, samples contained more Ti and less Si.

Tungsten deposits are associated with granite intrusions that record a long and complex evolution of a magnetic–hydrothermal system [50]. Due to regional metamorphism, segregation of minerals occurs in granites, which has been transformed into granite gneiss. These Himalayan rocks are exposed on the surface due to collisions of the Indian and Asian plates [51]. There is a possibility of Tungsten intrusion in astrophyllite mineral, which can be caused by the presence of W in the investigated sample. In the same way, appearance of other elements, Ag, Cr, Cu, and Ni, in the optical spectrum of astrophyllite might be due to impurities caused by regional metamorphism.

Existence of all the elements, except for Na, Li, and H, in astrophyllite sample, as detected by LIBS, was confirmed by XRF analysis. Reason for not detecting Na, Li and H lies in the insensitivity of XRF in the measurement of lighter elements. This is because the energy of the fluorescent X-rays depends on the atomic number of elements and energy levels of lighter elements, which have low energy that emits self-absorbed radiation by the sample. It is evident from Table 5 that the percentage error between the measurements of CF-LIBS and XRF techniques range from almost 8% to 18% for major elements (Ti, W, Fe), whereas for minor and trace elements, relative error increases from ±17% to ±88%. These results are consistent with many previous studies suggesting that CF-LIBS performs almost equivalently well for quantifying major elements in a multi-elemental material, but its efficiency reduces for minor and trace element quantification [25,26,27,28,29,30]. However, the advantage of LIBS over XRF is the detection of lighter elements in multi-elemental complex materials. Furthermore, the optical spectrum of a mineral can be obtained by focusing laser light directly on the surface of the mineral without any prior sample preparation, which is another advantage of LIBS over conventional analytical techniques.

There are many factors that hinder the quantitative efficiency of CF-LIBS technique including: (i) self-absorption; (ii) errors in measuring plasma parameters; (iii) errors in measuring spectroscopic parameters; (iv) errors in measuring the line intensities; (v) unavailability of suitable lines of some elements for quantitative analysis; (v) low ablation mass per laser pulse (≈ ng). It appears that for the purpose of measuring the amount of major elements in a chemically complex material, the CF-LIBS technique can be used as a reliable analytical method for practical purposes. However, for the accurate quantification of minor and trace elements, this technique requires further improvement on theoretical and instrumental fronts. There is need of development of software for theoretical calculations involved in CF-LIBS methodology to give quantitative results immediately. The use of a portable LIBS setup along with such software can be effective for on-site mineral exploration.

There is scarcity of matrix-matched reference materials for elemental analysis of geological materials with complex chemical composition. This fact limits the utility of the calibration method of LIBS for the purpose of compositional analysis of such materials. Being free from the matrix effect, CF-LIBS methodology emerges as an effective alternative and gives reasonable and good quantitative results for geological materials of unknown composition. In previous studies, quantitative results obtained with CF-LIBS showed a reasonable agreement with standard analytical techniques [26,27,28,29,30]. There is no need for reference samples and drawing the calibration curves for each element present in the target sample.

## 5. Conclusions

Compositional analysis of astrophyllite samples of local origin was performed using the calibration-free laser-induced breakdown spectroscopy (CF-LIBS) technique. Required plasma parameters for CF-LIBS calculations were measured, and conditions of local thermodynamic equilibrium (LTE) and optically thin plasma were verified. Overall, fifteen elements were identified in the emission spectrum of the sample, and ten out of the fifteen detected elements were quantified, including Ti, W, Fe, Cr, Cu, Ca, Mg, Ni, Si, and Al, having a concentration of 55.39%, 18.74%, 18.42%, 4.05%, 2.67, 0.35%, 0.18%, 0.12%, 0.06% and 0.02% by weight, respectively. However, the concentration of Na, Li, H, Cr, and Ag could not be calculated by the CF-LIBS method due to the unavailability of suitable spectral lines for analysis. An experiment performed by the X-ray fluorescence (XRF) method confirmed the presence of all the elements, which were detected by LIBS except for Na, H, and Li. Quantitative results of Ti, W, Fe, Cr, Cu, Ca, Mg, Ni, Si and Al as measured by XRF were 51.34%, 22.20%, 15.51%, 3.48%, 3.82%, 1.22%, 1.12%, 0.78%, 0.36% and 0.17% by weight, respectively. These quantitative values obtained by XRF are in reasonable agreement with those obtained by CF-LIBS. These quantitative results highlight the advantage that CF-LIBS method has, in quantification of multi-elemental geological materials, over the calibration method. In this methodology, drawing of curves for each element and requirement of a reference sample is avoided and still gives reasonable acceptable results. In general, this application of the CF-LIBS technique can be extended for the quantitative analysis of a range of geomaterials, and with some improvements, can be effectively used for mineral exploration. This research work might be of interest for the fraternity of scientists and engineers working in the area of geology, mineralogy, chemistry, and spectroscopy.

## Figures and Tables

**Table 1:** Spectral lines of various elements as identified in emission spectrum of astrophyllite.

Element	Wavelength (nm)
Ag-I	328.06
Ag-II	241.1,243.56, 274.39, 338.52
Al-I	305.97, 306.79, 396.67
Al-II	465.92
Ba-I	307.43, 350.1, 493.67, 553.54
Ba-II	455.2,455.63, 614.18, 649.81
Ca_I	393.59, 397.06, 442.92, 443.77, 445.33, 527.08, 558.86, 612.14, 616.22, 644.05, 646.33
Cr-I	425.34, 426.34, 427.48, 428.54, 476.42
Cu-I	324.93, 327.54, 515.34, 521.11
Cu-II	626.18,644.12
Fe-I	275.63, 309.10, 319, 372.17, 373.89, 374.33, 374.53, 375.11, 381.74, 382.19, 382.74, 383.6, 383.99, 384.25, 385.78, 386.13, 387.99, 388.43, 388.76,394.12,404.38,428.81, 438.46, 440.75,452.65, 456.65, 467.32, 490.77, 691.68
Fe-II	238.25, 240.55, 258.63, 259.97, 261.24, 274.03, 274.74, 275, 365.55, 327.37, 406.61, 431.77,439.77, 441.99, 444.64, 452.02, 500.22, 522.34, 506.78, 519.37 693.2
Li-I	610.35, 670.91
Mg-I	285.25, 516.84, 517.43, 518.44, 518.96
Mg-II	279.6, 280, 280.33
Na-I	588.98, 589.58
Ni-I	239.45, 344.43, 345.83, 346.35, 349.31, 351.2, 352.2, 352.82, 356.73, 357.17, 498.46, 499.37
Ni-II	239.45,
Si-I	250.78, 251.65, 252.97, 263.15, 288.22
Ti-I	307.68, 317, 323.04, 326.29, 332.43, 333.09, 333.65, 334.33, 336.24, 338.19, 338.91, 362.69, 363.33, 363.74, 364.41, 366.19, 366.4, 376.28, 399.2, 414.5, 416.63, 417.4, 430.85, 431.05,445.74, 449.61 451.27, 452.27, 453.47,461.95, 464.51, 468.19, 484.08, 488.87, 500.22, 500.96, 501.02, 503.88
Ti-II	308.37, 308.94, 311.76, 316.52, 320.6, 323.59, 323.76,325, 325.4, 328.85, 335.02, 337.40, 344.61, 364.95, 368.64, 376.06,447.08, 448.83 450.12, 462.51, 476.36, 487.4,
W-I	256.33, 272.82, 321.85, 322.42,375.56,382.81, 398.45, 400.14, 407.5, 429.33, 429.66, 432.84, 485.85, 446.01, 445.88, 449.17, 453.48, 551.41, 586.46
W-II	255.02,333.32, 408.12, 430.33

**Table 2:** Spectroscopic data of emission lines of Ti-I, Ca-I, and Fe-I used to draw Boltzmann plot.

Wavelength *λ* (nm)	Transitions	Statistical Weight		Transition Probability A (s^−1^)	Upper Level Energy *E_k_* (cm^−1^)
		$g_{k}$	$g_{i}$		
Ti (I) 323.59	3*d*^2^(^3^F)4*s*4*p*(^3^P°)→3*d*^2^4*s*(^4^F)5*d*	13	11	5.96 × 10^7^	48,106.65
Ti (I) 338.19	3*d*^3^(^4^F)4*s*→3*d*^3^(^2^H)4*p*	11	9	1.37 × 10^7^	41,341.54
Ti (I) 363.74	3*d*^2^4*s*^2^→3*d*^2^(^1^D)4*s*4*p*(^3^P°)	7	5	9.3 × 10^5^	27,480.06
Ti (I) 368.64	3*d*^2^4*s*^2^→3*d*^2^(^3^F)4*s*4*p*(^1^P°)	7	9	3.68 × 10^5^	27,498.98
Ti (I) 400.14	3*d*^2^(^3^F)4*s*4*p*(^3^P°)→3*d*^2^4*s*(^4^F)4*d*	7	9	1.36 × 10^7^	42,052.77
Ca (I) 445.33	3*p*^6^4*s*4*p*→3*p*^6^4*s*4*d*	7	5	8.70 × 10^7^	37,757.44
Ca (I) 612.14	3*p*^6^4*s*4*p*→3*p*^6^4*s*5*s*	3	3	2.87 × 10^7^	31,539.49
Ca (I) 646.33	3*p*^6^3*d*4*s*→3*p*^6^3*d*4*p*	7	5	4.70 × 10^7^	35,818.71
Fe (I) 240.55	3*d*^6^(^1^F)4*s*→3*d*^6^(^1^F)4*p*	8	8	2.46 × 10^8^	86,482.56
Fe (I) 373.86	3*d*^6^4*s*^2^→3*d*^6^(^3^G)4*s*4*p*(^3^P°)	7	5	3.44 × 10^7^	56,097.83
Fe (I) 438.62	3*d*^6^(^3^P2)4*s*→3*d*^6^(^5^D)4*p*	2	2	4.5 × 10^5^	45,206.47
Fe (I) 441.77	3*d*^6^(^3^P2)4*s*→3*d*^6^(^5^D)4*p*	4	2	2.1 × 10^5^	45,044.19

**Table 3:** List of spectral lines selected for electron number density calculation and calculated values.

Elements	Spectral Line Wavelength (nm)	$Ne=\frac{\Delta \lambda_{1/2}\times 10^{16}}{2\omega }\left(cm^{-3}\right)$
Ca-I	616.22 646.33	$5.82\times 10^{16}cm^{-3}$ $2.17\times 10^{16}cm^{-3}$
Si-I	251.65 288.18	$5.27\times 10^{16}cm^{-3}$ $2.41\times 10^{16}cm^{-3}$

**Table 4:** Spectroscopic parameters of selected emission lines of different compositional elements of astrophyllite sample used for calibration-free analysis.

Element.	$\mathrm{Wavelength}\;\lambda \;\left(nm\right)$	Transition Probability	Upper Level Statistical Weight (g_k_)	Lower Level Statistical Weight (g_i_)	Upper Level Energy *E_k_* cm^−1^
Cr-I	426.34	6.41 × 10^7^	17	15	54,498.25
Cr-I	428.87	2.80 × 10^6^	5	5	47,631.67
Cr-I	476.42	1.7 × 10^7^	9	7	49,620.56
Cu-I	515.34	6.00 × 10^7^	4	2	49,935.19
Cu-I	521.11	7.50 × 10^7^	4	6	63,584.65
Fe-I	319.00	3.07 × 10^7^	7	5	51,219.01
Fe-I	372.17	1.94 × 10^7^	5	5	51,370.14
Fe-I	373.89	3.44 × 10^7^	7	5	56,097.83
Fe-I	374.53	1.15 × 10^7^	5	7	52,916.29
Fe-I	381.74	4.16 × 10^6^	7	5	43,922.66
Fe-I	382.19	1.56 × 10^6^	11	11	47,834.55
Fe-I	383.6	3.29 × 10^7^	5	5	52,682.92
Fe-I	383.99	4.70 × 10^7^	4	5	34,017.10
Fe-I	385.78	7.25 × 10^6^	11	13	43,294.84
Fe-I	387.99	5.34 × 10^7^	3	3	52,180.82
Fe-I	394.12	9.10 × 10^6^	5	5	51,705.01
Fe-I	456.05	4.48 × 10^5^	3	5	48,516.13
Ti-I	338.91	7.71 × 10^6^	5	7	48,915.03
Ti-I	366.19	2.4 × 10^7^	7	7	45,893.19
Ti-I	366.4	2.45 × 10^7^	8	6	74,910.87
Ti-I	399.2	3.55 × 10^6^	1	3	41,871.36
Ti-I	488.87	1.34 × 10^7^	5	5	46,943.91
Ti-I	500.22	3.65 × 10^7^	3	5	45,093.22
W-I	256.33	1.14 × 10^7^	6	6	50,292.35
W-I	272.82	1.77 × 10^7^	5	7	55,619.66
W-I	321.85	3.0 × 10^6^	5	3	44,367.50
W-I	375.56	2.7 × 10^6^	9	7	54,911.61
W-I	398.45	1.39 × 10^7^	3	5	43,217.33
W-I	407.47	1.0 × 10^7^	7	7	27,488.11
W-I	446.01	2.93 × 10^6^	3	5	36,190.49
Ca-I	397.06	1.75 × 10^7^	3	5	40,474.24
Ca-I	445.33	8.7 × 10^7^	7	5	37,757.50
Ca-I	527.08	5.0 × 10^7^	5	7	39,340.08
Ca-I	558.86	4.9 × 10^7^	7	7	38,259.12
Ca-I	612.14	2.87 × 10^7^	3	3	31,539.50
Ca-I	616.22	4.77 × 10^7^	3	5	31,539.50
Ca-I	646.33	4.7 × 10^7^	7	5	35,818.71
Mg-I	516.84	1.13 × 10^7^	3	1	41,197.40
Mg-I	517.43	3.7 × 10^7^	3	3	41,197.40
Mg-I	518.96	5.61 × 10^7^	3	5	41,197.40
Si-I	263.15	1.06 × 10^8^	3	1	53,387.33
Si-I	288.22	2.17 × 10^8^	3	5	40,991.88
Al-I	309.28	1.16 × 10^7^	4	4	32,435.45
Al-I	396.15	9.85 × 10^7^	4	2	25,347.756

**Table 5:** Relative error of each element quantified by LIBS and XRF.

Element.	LIBS (wt.% Age)	XRF (wt.% Age)	Percentage Error (%)
Ti	55.39	51.34	±7.89
W	18.79	22.20	±15.36
Fe	18.30	15.51	±17.98
Cr	4.05	3.48	±16.38
Cu	2.66	3.82	±30.37
Ca	0.43	1.22	±64.75
Mg	0.18	1.12	±83.93
Ni	0.11	0.78	±84.61
Si	0.06	0.36	±83.33
Al	0.02	0.17	±88.23

## Data Availability

Data available on request due to restrictions eg privacy or ethical.The data presented in this study are available on request from the corresponding author.
